# Usefulness of Serum Cardiac Biomarkers for Predicting In-Hospital Cardiac Complications in Acute Hip Fracture: A Prospective Cohort in 20 High Surgical Risk patients with Age over 55 Years

**DOI:** 10.1155/2018/3453652

**Published:** 2018-07-05

**Authors:** Paphon Sa-ngasoongsong, Sorawut Thamyongkit, Noratep Kulachote, Kitchai Luksameearunothai, Tachapong Ngamukos, Chanyut Suphachatwong

**Affiliations:** ^1^Department of Orthopedics, Faculty of Medicine Ramathibodi Hospital, Mahidol University, Bangkok, Thailand; ^2^Chakri Naruebodindra Medical Institute, Faculty of Medicine Ramathibodi Hospital, Mahidol University, Bangkok, Thailand; ^3^Department of Orthopaedics, Faculty of Medicine Vajira Hospital, Navamindradhiraj University, Bangkok, Thailand; ^4^Department of Medicine, Faculty of Medicine Ramathibodi Hospital, Mahidol University, Bangkok, Thailand

## Abstract

*Background. *Serum cardiac biomarkers have recently been demonstrated to be useful for predicting perioperative complication after hip fracture (HF). However, no previous study has revealed the comparative efficacy of different cardiac biomarkers in high surgical risk HF patients.* Methods. *A prospective study was conducted, from June to December 2016, in 20 acute HF patients with American Society of Anesthesiologists (ASA) grade 3 or 4. All patients received blood test for high sensitivity Troponin-I (hsTnI) and N-terminal fragment of pro-B-type natriuretic peptide (NT-proBNP) at the time of admission and 24 hours postoperatively. Perioperative data and in-hospital, 3-month, and 6-month postoperative complications were collected. The complications were classified as cardiac and noncardiac HF-related complications.* Results. *The average patients' age was 79±8 years. Six patients (30%) were male. The incidence of PCI was 30% (n=6). None of the patients (0%) died during the 6-month postoperative followup period. In-hospital overall cardiac and noncardiac complications were found in 12(60%), 5(30%), and 7(45%), respectively. The mean serum hsTnI levels in the patients with cardiac complication were significantly greater than those in the patients without cardiac complication at both time of admission (99.5 ng/mL vs 5.5 ng/mL, p=0.006) and 24 hours postoperatively (28.6 ng/mL vs 9.4 ng/mL, p=0.013). The mean serum NT-proBNP levels in patients with cardiac complication were also greater but nonsignificantly compared to those in the patients without cardiac complication at both time of admission (2299 pg/mL vs 281 pg/mL, p=0.239) and 24 hours postoperatively (2266 pg/mL vs 586 pg/mL, p=0.061). The other significant preoperative predictors for cardiac complication were low hemoglobin level (p=0.014), low glomerular filtration rate level (p=0.039), and ASA grade 4 (p=0.005).* Conclusion. *In-hospital cardiac complication in high-risk HF patients was significantly associated with the abnormal rise of serum hsTnI level. Therefore, we recommended using the hsTnI test in the perioperative evaluation in high-risk HF patients.* Trial registration number* is TCTR20160711002.

## 1. Introduction

Hip fracture (HF) is a common fracture in the elderly population and is frequently associated with significant postoperative complications and mortality [1, 2]. Regarding the complications after HF, the cardiac complication is one of the most fearsome perioperative complications that accounts for 59.7% of the death during the first 48 hours postoperatively [2]. Moreover, the cardiac complication during the perioperative period is very common as an incidence of 35-42%, which is mostly caused by heart failure, myocardial ischemia, and arrhythmia [3]. Theoretically, this cardiac complication is a consequence of perioperative cardiac injury (PCI) that was triggered by many mechanisms related to fracture itself and the HF surgery, such as stress, blood loss, inflammation, hypercoagulation, or even in combination [4, 5]. However, the identification of PCI in HF patients is still problematic because PCI is often clinically unrecognized [6, 7] and the diagnosis requires the measurement of serum cardiac biomarkers, such as such as N-terminal fragment of pro-B-type natriuretic peptide (NT-proBNP) and cardiac troponin. Recent studies showed that the increase of these cardiac biomarkers during admission is significant predictor for perioperative cardiovascular complication [8–14]. However, only few previous studies had been investigated on the relationship between the abnormal rise in cardiac biomarkers and the development of the complication after HF during perioperative period [14, 15]. Moreover, to our best knowledge, the comparative data between the efficacy of the perioperative assessment with different cardiac biomarkers for predicting the in-hospital or early postoperative morbidity or mortality has not been studied yet. Therefore, the aim of this study was to evaluate and compare the efficacy of the two commonly used cardiac biomarkers, high sensitivity Troponin-I (hsTnI), and NT-proBNP, for predicting the complication after HF during the in-hospital and 6-month postoperative period.

## 2. Materials and Methods

### 2.1. Study Population

This was a prospective single-centered observational cohort study in an academic university hospital from June to December 2015 in older patients with acute hip fracture. The study protocol was approved by our Ethical Clearance Committee on Human Rights Related to Research Involving Human Subjects, Faculty of Medicine Ramathibodi Hospital, Mahidol University (protocol number 09-58-15). All patients were informed and gave their consent before participating in this study. The inclusion criteria were patients who were (1) 55 years or older, (2) presented with hip fracture within 1 week before admission, (3) treated with surgery, and (4) having American Society of Anesthesiologist (ASA) physical status grade 3 or 4. Patients were excluded if they (1) had serum creatinine >2.0 mg/dL, (2) died before surgery, (3) received neuropeptides therapy, and (4) had severe dementia or were uncooperative for assessments. All participants were followed postoperatively for 6 months.

### 2.2. Surgical Protocol and Perioperative Management

After the patients were diagnosed as hip fracture and gave their informed consent, they were allocated into this study and admitted to the orthopaedic trauma ward. Then preoperative medical consultation and surgical planning were performed. The surgery was scheduled as soon as possible after the medical clearance, and all operations were performed by one of the orthopaedic trauma experts (PS and NK). Displaced femoral neck fractures were treated with bipolar hip replacement (BHR) using anterolateral approach with anterior hemimyotomy. Prosthesis selection, as cementless or cemented type, was based on the quality of proximal femoral geometry. Dynamic hip screw (DHS) was used in stable intertrochanteric fracture, and proximal femoral nail antirotation (PFNA) was used in unstable intertrochanteric fracture. All patients received blood test for high sensitivity Troponin-I (hsTnI) and NT-proBNP on the admission day and 24 hours postoperatively. NT-proBNP and hsTnI were measured by electrochemiluminescence immunoassay on a Dimension Vista 500 (Siemens Healthcare Diagnostics, Deerfield, Illinois, US). The normal value of hsTnI was defined as less than 34.2 ng/mL in males and 15.6 ng/mL in females [16]. The same postoperative protocol was applied to all patients, with early ambulation as soon as possible. Intermittent pneumatic pump was applied to all patients. Blood transfusion was considered when hemoglobin (Hb) was less than 8 g/dL or the patients had anemic symptoms (dyspnea, tachypnea, and hypoxemia). Controlled weight bearing ambulation on the injured leg with gait aid was allowed regarding the operation performed. Partial weight bearing was used for DHS, PFNA, and cementless BHR and then progress to full weight bearing after 6 weeks postoperatively. The patients who underwent cemented BHR were allowed postoperative full weight bearing with gait aid. Postoperative outcome and complications were followed for 6 months.

### 2.3. Data Collection

Demographic and perioperative data, including age, gender, body mass index, fracture type and side, comorbid disease, ASA physical status, operation, intraoperative blood loss, and postoperative length of stay (PLOS) were recorded. Age and comorbid diseases were then calculated into Charlson comorbid index (CCI). Preoperative laboratory values, including hemoglobin (Hb), glomerular infiltration rate (GFR), and serum albumin, were collected. Postoperative complications were classified into cardiac and noncardiac complications. Cardiac complication was defined as any of cardiovascular adverse events such as myocardial infarction (MI), congestive heart failure (CHF), or new-onset cardiac arrhythmia in the patients without underlying cardiac arrhythmia or uncontrolled cardiac arrhythmia in the patients with pre-existing cardiac arrhythmia, unstable angina, or death from cardiac complication. CHF was then classified as heart failure with preserved ejection fraction (HFpEF) and heart failure with reduced ejection fraction (HFrEF). Noncardiac complication was defined as any of noncardiovascular adverse events, such as pressure ulcer, infection, delirium, pulmonary complication, acute renal failure (ARF), deep vein thrombosis (DVT), pulmonary embolism (PE), or death from noncardiac complication. The primary outcome was the in-hospital cardiac complication, and the secondary outcomes were in-hospital noncardiac events as mentioned above.

### 2.4. Statistical Analysis

Normally distributed continuous data were shown as mean ± standard deviation (SD) and compared using student's t test. Nonnormally distributed continuous data were shown as median (interquartile range [IQR]) and compared using the Mann–Whitney* U* test. The categorical variables were presented as number of cases with proportion and compared with Chi-square or Fisher's exact test, as appropriate. A* p* value<0.05 was considered statistically significant. The efficacy of the serum cardiac biomarkers for predicting the in-hospital cardiac complication and the cut-off reference level for NT-proBNP were assessed by the Receiver-Operator Characteristic (ROC) curve analysis. The difference between the risk factors for cardiac complication was assessed by the relative risk (R.R.) and their 95% confidence interval (CI). Statistical analysis was performed using the SPSS for Windows v15.0 (SPSS Inc., Chicago, IL, USA) software.

## 3. Results

**Figure 1:**
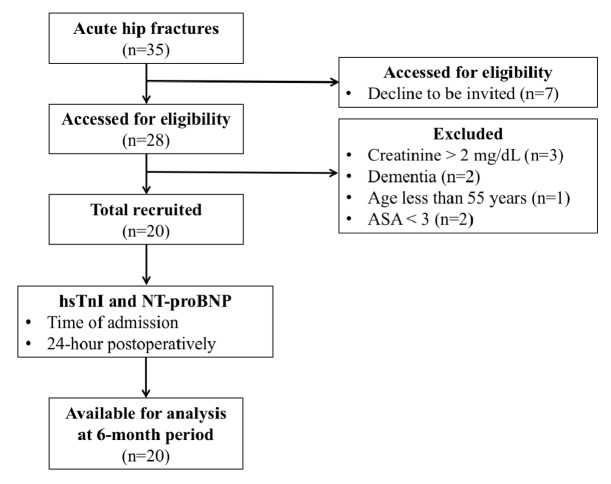
Flow diagram of this study.

A total of 20 acute hip fracture patients were included in this study (Figure 1).  Table 1 demonstrated the baseline characteristic data of the study population. The mean patients' age was 79 years (range: 55-90 years) and 14 (70%) of them were females. The fractures were classified as femoral neck fractures in 9 patients (45%) and intertrochanteric fractures in 11 patients (55%). Patients who had femoral neck fractures were treated with either cemented (n=5) or cementless (n=4) bipolar hip replacements. Patients with unstable intertrochanteric fractures (n=9) were treated with PFNA, and those who had stable intertrochanteric fractures (n=2) were treated with DHS. The most common comorbid disease was hypertension (70%), and the pre-existing ischemic heart disease was found in 4 patients (20%). The mean CCI was 4.6 ± 1.4. The mean time from fracture to surgery was 2.3 days (range: 1-6 days).

Postoperative complications during the 6-month followup period was shown in Table 2. The incidences of overall cardiac and noncardiac complications during admission (in-hospital period) were all significantly higher compared to the other periods during the 6-month followup after discharge (*p*=<0.0001, 0.033, and 0.009, respectively). During the perioperative period, cardiac complications occurred in 5 patients (1 preoperative HFpEF, 1 preoperative unstable angina, 1 postoperative HFpEF on day 2, 1 postoperative AF with RVR on day 3, and 1 postoperative AF and RVR with HFrEF). Noncardiac complications occurred in 7 patients (1 with combined ARF, UTI, and delirium, 1 had ARF and delirium, 1 ARF and DVT, 1 DVT, 1 PE, and 2 UTI), respectively. All patients had survived during the 6-month followup period.

Table 3 presented the comparison of the serum level of hsTnI and NT-proBNP, at the time of admission and 24 hours postoperatively, between the patients who had and did not have cardiac or noncardiac complications. Regarding the cardiac complication, the mean hsTnI levels in the patients having cardiac complication were significantly greater than those in the patients not having cardiac complication at both the time on admission (99.5 ng/mL vs 5.5 ng/mL,* p*=0.006) and 24 hours postoperatively (28.6 ng/mL vs 9.4 ng/mL,* p*=0.013). The mean NT-proBNP levels in the patients having cardiac complication were greater than those in the patients who not having cardiac complication at both time points, but they did not reach the statistical significance (on admission: 2299 pg/mL vs 281 pg/mL,* p*=0.239, and 24 hours postoperatively: 2266 pg/mL vs 586 pg/mL,* p*=0.061).

Regarding the noncardiac complication, the mean serum hsTnI levels in the patients who had noncardiac complication did not significantly differ from those in the patients who did not have noncardiac complication at both time on admission (8.4 ng/mL vs 9.5 ng/mL,* p*=0.843) and 24 hours postoperatively (9.0 ng/mL vs 10.5 ng/mL,* p*=0.501). Also, there was no significant difference between the mean NT-proBNP levels in the patients who had and did not have noncardiac complication at both time on admission (331 pg/mL vs 781 pg/mL,* p*=0.362) and 24 hours postoperatively (586 pg/mL vs 821 pg/mL,* p*=0.905) (Table 3).

**Figure 2:**
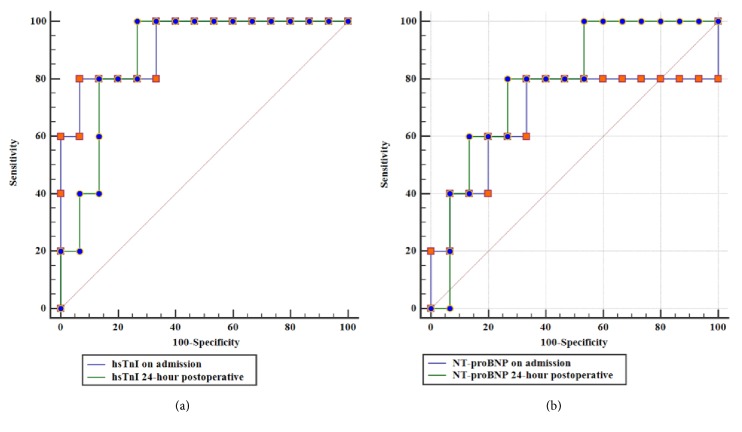
ROC analysis of using (a) hsTnI and (b) NT-proBNP, at the time on admission and 24 hours postoperatively, for predicting the in-hospital cardiac complication.

Figure 2 illustrated the ROC analysis on using serum cardiac markers for predicting in-hospital cardiac complication.  Figure 2(a) revealed that using the serum hsTnI test, at both the time on admission and 24 hours postoperatively, had significant association for predicting this complication (*p*<0.0001 both) with very good accuracy. The area under the curve (AUC) of hsTnI at the time on admission and 24 hours postoperatively was 0.920 (95% CI: 0.709-0.993) and 0.880 (95% CI: 0.658-0.981), respectively. However, the NT-proBNP test was significantly associated with the prediction of this complication only with those at 24 hours postoperatively (*p*=0.013) (*p* value from using the NT-proBNP test at the time on admission = 0.334). The AUC of NT-proBNP at the time on admission and 24 hours postoperatively were 0.680 (95%CI: 0.437-0.867) and 0.787 (95%CI: 0.549-0.935). With the cut-off level for the NT-proBNP at 24 hours postoperatively as 821 pg/mL, this would result in a sensitivity of 80%, and a specificity of 73% (Figure 2(b)).

The correlation between the patients' characteristics and in-hospital postoperative complication was shown in Table 4. Regarding the in-hospital cardiac complication, the significant factors that were associated with this complication were ASA status grade 4 (*p*=0.005), preoperative Hb level (*p*=0.014), GFR (*p*=0.039), and the abnormal high serum hsTnI level at the time on admission (*p*=0.005) and at 24 hours postoperatively (*p*=0.014). The patients who had abnormal high serum hsTnI level, at the time of admission or 24 hours postoperatively, had 12 and 6 times greater risk for developing the in-hospital cardiac complication compared to those who had normal serum hsTnI level. Additionally, some factors, such as age, CCI, and serum albumin level, also showed a tendency toward cardiac complication, but these did not reach a significant effect (p=0.083, 0.056, and 0.059, respectively). Concerning the in-hospital noncardiac complication, the significant factors associated with this complication were the time from fracture to surgery (p=0.005), ASA status grade 4 (*p*=0.031), and postoperative length of stay (PLOS) (*p*=0.034) (Table 4).

## 4. Discussion

Perioperative cardiac injury (PCI) is common among the hip fracture patients, especially in those who are ageing population and having multiple comorbid diseases. Early PCI diagnosis is important and beneficial for risk stratification and appropriate management in for each individual patient. Recently, several studies have demonstrated a significant correlation between the increase of serum cardiac biomarkers level and the poor outcome after noncardiac surgery. However, only few studies have demonstrated the association between the abnormal rise of cardiac biomarkers and the perioperative complication after hip fracture [14, 15]. This study aimed to assess and compare the efficacy of two cardiac biomarkers, hsTnI and NT-proBNP, for predicting the in-hospital, 3-month, and 6-month postoperative complication after HF.

Our study showed that the PCI, as shown by the abnormal rise of serum hsTnI level, was common as an incidence of 30% (n=6) in the HF patients and it could be detected as early as at the time of admission (Table 3), which was comparable to the previous studies [14, 15]. These results could imply that the HF itself and the poor physical condition of the HF patients are the major initiate factors for the PCI and should be responsible for the cardiac complication in preoperative period [17]. Also, the HF surgery could be the second factor that further stimulates the PCI resulting in the postoperative cardiac complication. The findings of this study also demonstrated that the abnormal rise of serum hsTnI, at either time of admission or 24-hours postoperatively, was significantly associated with the in-hospital cardiac complication and, therefore, was useful as one of the significant predictive factors for the in-hospital cardiac complication, the same as the other clinical predictive factors (ASA grade 4, Hb, and GFR). The results of the present study were comparative with those of previous studies that serum cardiac troponin test in elderly hip fracture has prognostic significance for postoperative mortality and morbidity [15, 18, 19]. However, none of these patients died during the 6-month postoperative period, so we could not find any association with the abnormal rising test and postoperative mortality. This may be explained by the facts that our study was a prospective study with strict protocol on perioperative management and early detection of those complications. As a result, our results on mortality and morbidity, especially from cardiac complication, may be better than those previous reports.

Regarding the NT-proBNP test for PCI diagnosis, the results of this study showed that the mean serum NT-proBNP level was also increased, the same as the hsTnI, but nonsignificantly in the patients who had in-hospital cardiac complication compared to those who did not have (Table 3). This might be explained by the small sample size of the present study. However, these findings were comparable to the previous studies that the prognostic information from the NT-proBNP was not as strong as those from the high sensitive cardiac troponin test [20]. Moreover, an increase of the NT-proBNP might be found in those who had a transient myocardial ischemia [21] or who received considerable infusion of intravenous fluid [22], and these could result in a lower diagnostic accuracy and the need of a high cut-off reference value [23–25]. This study also showed that the prognostic value of the NT-proBNP might be better in postoperative period (p=0.061) compared to the preoperative use (p=0.239) (Table 3), and this finding was comparable to the previous studies that recommended the use of NT-proBNP in the postoperative period and outpatients clinic setting [26].

The results from this study also demonstrated that in-hospital noncardiac complication was significantly associated with some factors as the time from fracture to surgery (p=0.005), ASA grade 4 (p=0.031), and postoperative length of stay (p=0.034). These findings were comparable with the previous studies on hip fracture [2, 3, 12, 15, 23, 27] and, therefore, highlighted the importance of the preoperative risk stratification and proper management on these high surgical risk patients.

This study also had some limitations. First, the study population was relatively small due to the nature of our prospective study and included only high-risk HF patients. Therefore, this may not detect other significantly clinical outcomes, such as the effect of serum cardiac biomarkers on postoperative mortality. Second, we excluded the patients who had creatinine ≥ 2 mg/dl to avoid the false positive value of abnormal rising test from very poor renal function. Thus, our results might not be applied to the patients with pre-existing severe renal disease. Lastly, the baseline serum cardiac biomarkers level before HF was not available in this study which would result in a selection bias and affected the outcome. Therefore, further prospective studies with larger study population are required for better clarification.

## 5. Conclusion

Our study showed that in-hospital cardiac complication in high-risk HF patients was significantly associated and predictable with the abnormal rise of serum hsTnI level, which is the same as the other significant predictive factors such as low preoperative Hb level, poor renal function, and poor physical status. We recommend using hsTnI for risk stratification during the perioperative period of HF surgery on high surgical risk patients.

## Figures and Tables

**Table 1:** Characteristics of study population.

**Patients' characteristics**	**Value**
Age, year *▯*	79 ± 8
Male gender ◆	6 (30)
Body mass index, kg/m^2^*▯*	21.1 ± 3.7
Femoral neck fracture: Intertrochanteric fracture	9 : 11
Injury on right side ◆	9 (45)
ASA physical status grade 3 : 4	15 : 5
Comorbid diseases ◆	
Diabetes	4 (20)
Hypertension	14 (70)
COPD	4 (20)
Ischemic heart disease	4 (20)
Atrial fibrillation	3 (15)
History of stroke	4 (20)
History of malignancy	2 (10)
Charlson comorbidity index *▯*	4.6 ± 1.4
Preoperative laboratory investigations *▯*	
Hemoglobin, g/dL	10.8 ± 2.0
GFR, mL/minute/1.73 m^2^	65.4 ± 24.3
Albumin, g/L	32.3 ± 4.2
The time from fracture to surgery, day *▯*	2.3 ± 1.5
Intraoperative blood loss, mL *▯*	284 ± 282

*▯*: values presented as mean ± standard deviation.

◆: values presents as number of cases with those condition (percentage).

COPD: chronic obstructive pulmonary disease.

GFR: glomerular infiltration rate.

**Table 2:** In-hospital complications and postoperative complications after discharge during 6-month followup period.

**Complications**	**In-hospital**	**0-3 months**	**4-6 months**	***p*-value**
Overall ◆	12	2	0	**<0.0001** **∗**
Cardiac complications ◆				
Total	5	1	0	**0.033** **∗**
CHF	3	1	0	0.647
Cardiac arrhythmia	2	0	0	
Unstable angina	1	0	0	
Non-cardiac complications ◆				
Total	7	1	1	**0.009**
Infection	3	1	1	0.536
Renal	3	0	0	
VTE	3	0	0	
Delirium	2	0	0	

◆: no. of patients having that complication; *∗*: significant difference with *p*<0.05.

CHF: congestive heart failure; VTE: venous thromboembolism.

**Table 3:** Comparison of the serum level of cardiac biomarkers between the patients who had and did not have either cardiac and noncardiac complications, at the time on admission and 24 hours postoperatively.

	**Total (n=20)**	**Cardiac complication**		*p*-value	**Non-cardiac complication**		*p*-value
		**Yes (n=5)**	**No (n=15**)		**Yes (n=7)**	**No (n=13)**	
**hsTnI, ng/mL ** **⌘**							
On admission	8.6 (5.2, 22.0)	99.5 ± 112.9	5.5 (5.0, 10.6)	**0.006** **∗**	8.4 (5.5, 154.4)	9.5 (5.0, 20.0)	0.843
Postoperative 24 hr	10.3 (8.2, 23.8)	28.6 (17.0, 461,3)	9.4 (7.9, 12.8)	**0.013** **∗**	9.0 (6.9, 99.0)	10.5 (9.2, 21.4)	0.501
**NT-proBNP, pg/mL ** **⌘**							
On admission	356 (214, 1643)	2299 ± 2629	281 (208, 802)	0.239	311 (132, 456)	781 (231, 1923)	0.362
Postoperative 24 hr	644 (389, 1830)	2266 ± 1677	586 (310, 943)	0.061	586 (516, 1937)	821 (318, 1661)	0.905

hsTnI: high sensitivity troponin I; NT-proBNP: N-terminal fragment of pro-B-type natriuretic peptide.

*⌘*: normally distributed value presented as mean standard deviation, and abnormally distributed value presented as median (interquartile range).

*∗*: significant difference with *p* < 0.05.

**Table 4:** The relationship between patients' characteristics and in-hospital complications.

	**Cardiac complication**				**Non-cardiac related complication**			
	**Yes (n=5)**	**No (n=15)**	***p*-value**	**RR (95% C.I.)**	**Yes (n=7)**	**No (n=13)**	***p*-value**	**RR (95% C.I.)**
Age, year	84 ± 3	77 ± 9	0.083		78 ± 10	79 ± 7	0.803	
Male gender	1 (20)	5 (33)	1	0.58 (0.08-4.19)	2 (29)	4 (31)	1	0.93 (0.22-3.87)
BMI, kg/m2	20.1 ± 4.3	21.4 ± 3.6	0.499		21.7 ± 4.6	20.8 ± 3.3	0.615	
Intertrochanteric fracture	3 (60)	8 (53)	1	1.23 (0.26-5.82)	4 (57)	7 (54)	1	1.06 (0.47-2.40)
Injury on right side	3 (60)	6 (40)	0.617	1.83 (0.39-8.70)	3 (43)	6 (46)	1	0.93 (0.33-2.62)
Time from fracture to surgery, day	2.8 ± 1.9	2.1 ± 1.3	0.352		3.4 ± 1.9	1.6 ± 0.7	**0.005** **∗**	
ASA grade 4	4 (80)	1 (7)	**0.005** **∗**	12 (1.72-83.8)	4 (57)	1 (8)	**0.031** **∗**	7.43 (1.02-54.3)
Comorbid disease								
DM	1 (20)	3 (20)	1.000	1.00 (0.15-6.67)	1 (14)	3 (23)	1.000	0.62 (0.08-4.90)
Hypertension	3 (60)	11 (73)	0.613	0.64 (0.14-2.91)	4 (57)	10 (77)	0.613	0.73 (0.37-1.51)
IHD	2 (40)	2 (13)	0.249	2.67 (0.65-10.97)	1 (14)	3 (23)	1.000	0.62 (0.08-4.90)
AF	2 (40)	1 (7)	0.140	3.78 (1.03-13.89)	1 (14)	2 (15)	1.000	0.93 (0.10-8.53)
COPD	1 (20)	3 (20)	1.000	1.00 (0.15-6.67)	1 (14)	3 (23)	1.000	0.62 (0.08-4.90)
Stroke	0 (0)	4 (27)	0.530	0.31 (0.02-4.68)	1 (14)	3 (23)	1.000	0.62 (0.08-4.90)
Malignancy	1 (20)	1 (7)	0.447	2.25 (0.44-11.52)	1 (14)	1 (8)	1.000	1.86 (0.14-25.4)
CCI	5.6 ± 1.3	4.2 ± 1.3	0.056		4.3 ± 1.6	4.7 ± 1.4	0.559	
Hemoglobin, g/dL	8.9 ± 1.8	11.4 ± 1.7	**0.014** **∗**		10.8 ± 2.6	10.7 ± 1.7	0.955	
GFR, mL/minute/1.73m^2^	46.3 ± 19.3	71.7 ± 22.8	**0.039** **∗**		55.8 ± 29.7	70.5 ± 20.3	0.204	
Albumin, g/L	29.3 ± 4.0	33.3 ± 3.9	0.059		32.6 ± 3.3	32.2 ± 4.8	0.838	
Intraoperative blood loss, mL	450 ± 499	228 ± 154	0.383	1.64 (0.53-5.09)	200 (150, 338)	300 (50, 363)	0.968	
PLOS, day	10 (3, 15)	4 (3, 5)	0.172		7 (4, 10)	3 (3, 5)	**0.034** **∗**	
Positive hsTnI with normal cut-off reference level								
On admission	4 (80)	1 (7)	**0.005** **∗**	12.0 (1.7-83.8)	2 (29)	3 (23)	1.000	1.24 (0.27-5.75)
Postoperative 24 hour	4 (80)	2 (13)	**0.014** **∗**	6.0 (1.54-23.4)	2 (29)	4 (31)	1.000	0.93 (0.22-3.87)
Positive NT-proBNP with cut-off level as 821 pg/mL								
On admission	3 (60)	3 (20)	0.131	3.0 (0.87-10.36)	1 (14)	5 (38)	0.354	0.37 (0.05-2.59)
Postoperative 24 hour	4 (80)	4 (27)	0.109	3.0 (1.16-7.73)	2 (29)	6 (46)	0.642	0.62 (0.17-2.29)

BMI: body mass index; ASA: American Society of Anesthesiologist; DM: diabetes milletus; IHD: ischemic heart disease; AF: atrial fibrillation.

COPD: chronic obstructive pulmonary disease; CCI: Charlson comorbidity index; PLOS: postoperative length of stay; GFR: glomerular infiltration rate.

*▯*: values presented as mean ± standard deviation. ◆: values presented as number of cases (percentage); ■: values presented as median (interquartile range).

*∗*: significant difference with *p*<0.05

## Data Availability

Technical appendix, statistical code, and dataset are available from the corresponding authors at noratep28@gmail.com.
